# A 3D Bioprinted Pseudo-Bone Drug Delivery Scaffold for Bone Tissue Engineering

**DOI:** 10.3390/pharmaceutics12020166

**Published:** 2020-02-17

**Authors:** Pariksha Jolene Kondiah, Pierre P. D. Kondiah, Yahya E. Choonara, Thashree Marimuthu, Viness Pillay

**Affiliations:** Wits Advanced Drug Delivery Platform Research Unit, Department of Pharmacy and Pharmacology, School of Therapeutic Sciences, Faculty of Health Sciences, University of the Witwatersrand, Johannesburg, 7 York Road, Parktown 2193, South Africa; parikshakondiah@gmail.com (P.J.K.); pierre.kondiah@wits.ac.za (P.P.D.K.); yahya.choonara@wits.ac.za (Y.E.C.); Thashree.marimuthu@wits.ac.za (T.M.)

**Keywords:** 3D bioprinting, polymeric ink, optimization, pseudo-bone, implantable scaffold, computer-aided design (CAD) design, drug delivery

## Abstract

A 3D bioprinted pseudo-bone drug delivery scaffold was fabricated to display matrix strength, matrix resilience, as well as porous morphology of healthy human bone. Computer-aided design (CAD) software was employed for developing the 3D bioprinted scaffold. Further optimization of the scaffold was undertaken using MATLAB^®^ software and artificial neural networks (ANN). Polymers employed for formulating the 3D scaffold comprised of polypropylene fumarate (PPF), free radical polymerized polyethylene glycol- polycaprolactone (PEG-PCL-PEG), and pluronic (PF127). Simvastatin was incorporated into the 3D bioprinted scaffolds to further promote bone healing and repair properties. The 3D bioprinted scaffold was characterized for its chemical, morphological, mechanical, and in vitro release kinetics for evaluation of its behavior for application as an implantable scaffold at the site of bone fracture. The ANN-optimized 3D bioprinted scaffold displayed significant properties as a controlled release platform, demonstrating drug release over 20 days. The 3D bioprinted scaffold further displayed formation as a pseudo-bone matrix, using a human clavicle bone model, induced with a butterfly fracture. The strength of the pseudo-bone matrix, evaluated for its matrix hardness (MH) and matrix resilience (MR), was evaluated to be as strong as original bone, having a 99% MH and 98% MR property, to healthy human clavicle bones.

## 1. Introduction

3D bioprinting is currently the most explored field of research in mechanical microenvironment tissue-engineered systems [1]. The most common impairments employing 3D bioprinting research significantly focus on therapeutics designed to treat bone fractures as well as bone defects [2]. Consequently, bone-related costs and therapy are escalating [3]. Typical treatment for these impairments includes bone grafts or metal prosthetic implants. However, this form of therapy is restricted in many incidents due to significant tissue loss resulting from surgery, long recovery periods, and donor site morbidity [4]. Nonetheless, the limitations related to these forms of therapy have opened doors to the evolution of 3D bioprinting technology, employing cutting edge design and execution of drug delivery engineered platforms [5]. Bone tissue repair and regeneration employing noninvasive procedures have become a significant focus due to the implementation of 3D bioprinting technology [6,7,8].

3D bioprinted scaffolds employed for bone tissue repair and healing, using computer-aided design (CAD) software, has numerous benefits such as intensive care patient-specific dose designs, customized geometrical site-specific drug delivery applications, as well as controlled drug release implantable platforms employing internal architecture modification designs [9,10]. To date, 3D printing has gained superior recognition in the industrial market, ranging from medical devices, engineering components to pharmaceutical drug technologies [11,12,13,14]. Previous studies conducted in 3D printing and bone tissue engineering have employed polymers of similar nature, designed to strengthen scaffold conformation designs, as well as obtain suitable bioinks/ polymeric inks, for a variety of printing architecture. Studies have reported hydrogel formulations for bone support (injectable hydrogels), bone regeneration using natural (chitin and alginate derivatives) and synthetic polymers (silicone and inorganic complexes), various 3D inkjet bio-fabrications, as well as incorporation of growth factors within porous scaffold designs [10,11,12,13,14,15,16,17]. Biomineralization, osteogenesis, and hard tissue promoting therapeutics have also been an area of significant focus due to exponential cases of accident-related bone fractures and osteoporotic pathologies. However, an essential component of such research should provide a means of improving the cost-effectiveness of products, thereby causing minimum wastage and time efficiency over the synthetic process. Nevertheless, this will also require highly accurate batches when undertaking 3D bioprinting, so as to achieve reproducible therapeutic delivery systems [15,16,17,18].

In this study, we synthesized a pseudo-bone drug delivery scaffold, possessing properties of comparable matrix hardness and resilience to healthy bone tissue, following in situ analysis. This study follows as a trajectory from previous pre-formulation studies undertaken [19], with further optimization employing 3D bioprinting for advanced drug delivery. The 3D bioprinted pseudo-bone scaffold formulations were designed using polymer-variable concentration optimization, employing MATLAB^®^ software (MathWorks^®^, Natick, MA, USA) and artificial neural networks. The 3D bioprinted scaffold was designed using Inventor^®^ (Autodesk^®^, San Rafael, CA, USA) auto CAD, fabricating a strategic cylindrical-shaped drug delivery scaffold, with uniform bioprinted filaments and pore size geometrical configuration. The pseudo-bone 3D bioprinted scaffold was designed to mimic the morphology, matrix strength, and matrix resilience of healthy human bone. This was evaluated using healthy human clavicles in which butterfly fractures were induced. Ethical clearance waiver was granted from the Department of Human Anatomy, University of the Witwatersrand, for undertaking this study (ethics clearance number: W-CJ-140604-1; granted date: 3 July 2018).

This drug delivery system was optimized as a controlled release platform incorporating the drug simvastatin. The polymeric ink was designed to gradually degrade and release its loaded contents in a sustained manner, allowing contact adhesion between fractured/damaged bone and formation of a pseudo-bone matrix within these sites. Polymers employed for formulating the polymeric ink of the pseudo-bone scaffold consisted of polypropylene fumarate (PPF), free radical polymerized polyethylene glycol-polycaprolactone (PEG-PCL-PEG), and Pluronic (PF127). The 3D bioprinted scaffold was characterized for its chemical, morphological, mechanical, and in vitro release properties and optimization design using MATLAB^®^ software and artificial neural networks. 

The analysis of a designed multilayered network, using a feed-forward backpropagation, with multiple inputs, outputs, as well as varied hidden layers, was designed. This system comprised of multilayer nonlinear networks. In the design, a back-propagation relationship using large input and output datasets, determining network mapping, thereby not requiring a definite mathematical equation to undertake the modeling, was achieved. This model, employing a gradient descent algorithm using backpropagation, was classified as the Widrow–Hoff learning rule, using multiple-layer networks, with various degrees of optimization to the algorithm.

In this study, the 3D bioprinted scaffolds, designed and evaluated as 39 formulations using MATLAB^®^ software, comprised of variables of PPF (8% *w*/*v*–20% *w*/*v*) and PF127 (14% *w*/*v*–16% *w*/*v*). Results presented from these design scaffold formulations were studied in response to duration of release of simvastatin and the degree of thermogelation of the polymeric ink formulation. Analysis was undertaken determining the formulation compositions and response factor from each design, using a 3D Simulink design graph. The release analysis of further evaluating the relationship between the hydrophobic chain regions of PPF encapsulating simvastatin was undertaken, correlating the duration of drug release from the 3D bioprinted scaffold over time. 

## 2. Materials and Methods 

### 2.1. Materials 

PEG (Mw 4000), stannous octoate, 92.5%; Pluronic F-127; poly(ethylene glycol) diacrylate; epsilon-caprolactone, 99%; petroleum ether, 90%; and simvastatin (molecular weight: 418.57), 97% purity, were procured from Sigma-Aldrich (St. Louis, MO, USA). Methanol, 99%; diethyl fumarate, 98%; dichloromethane, diethyl ether (anhydrous); hydroquinone, 99% purity; methylene chloride; propylene glycol (1,2-propandiol); hydrochloric acid, 1.85% *v*/*v*; sodium sulphate; and zinc chloride were purchased from Merck (Pty) Ltd. (Modderfontein, South Africa). All software employed in this study was procured from EnvisionTEC^®^ GmbH (Gladbeck, Germany). All other reagents were of analytical grade and were employed as received. All reactions were undertaken under inert conditions.

### 2.2. Synthesis of the Polymeric Ink Formulation

A strategically designed copolymeric blend of polymers, polypropylene fumarate (PPF), PEG-PCL-PEG, and pluronic PF 127, was optimized for 3D bioprinting and loaded with simvastatin drug. Free radical polymerization was undertaken for preparation of copolymer PEG-PCL-PEG using PEG (Mw 4000) as the macroinitiator and catalyst stannous octoate (Sn(Oct)_2_). Briefly, 0.007 M of PEG 4000 and 0.098 M of ε-caprolactone was reacted in a round bottom flask, purged with nitrogen, at a temperature of 125 °C, under constant magnetic stirring (3500 rpm). The catalyst (100 μL) was then added to the reaction and left for 6 h under nitrogen purging. PPF (8% *w*/*v*–20% *w*/*v*) and PF 127 (14% *w*/*v*–16% *w*/*v*) were then added to the reaction mixture, specifying the concentrations as obtained by the designed formulations using MATLAB^®^ software. The reaction temperature was then increased to 140 °C and left for 6 h under constant magnetic stirring of 3000 rpm. The reaction mixture was then allowed to cool to room temperature, with further addition to Dichloromethane (DCM), and washing thrice with deionized water. The organic solvent was then removed using rotary evaporation and stored at 10 °C for further use. Details of the above synthesis have previously been reported by authors [19]. Simvastatin was then loaded into the copolymer, with a therapeutic dose calculated at 10 mg per scaffold. The dose for loading was calculated according to the material required for bioprinting, dependent on parameters employed, according to the optimization of fabrication procedures on the bioprinted scaffold, as discussed in Section 2.4. The polymeric ink paste was then fabricated by formulating a ratio of 6:3:1 of the copolymer: methanol: deionized water, respectively. The copolymer then underwent microwave-assisted heating using a specific laboratory designed MAS-II Plus Microwave Synthesis Workstation (Sineo, China) at 50 °C for 10 min, at 600 W. Distilled water was added to the polymeric ink (polymeric ink: water ratio; 6.5:3.5) for its application of drug loading and release analysis. Ten milligrams of drug was loaded in the polymeric ink at a temperature of 10 °C for 6 h. Thereafter, the loaded polymeric ink was incubated at 25 °C for 2 h to ensure maximum drug loading occurred during the gelling phase. The loading of drug was back-calculated according to sample volume in each printing cartridge such that 10 mg of simvastatin was present in a total of 7 layers of the 3D printed scaffold.

### 2.3. Artificial Neural Network Design and Optimization of the 3D Bioprinted Scaffold

Artificial neural networks (ANN) can be used to determine linear and nonlinear sophisticated relationships between dependent and independent variables in a study [20]. The fundamental benefit of using ANN is the capacity for the neural network to learn directly from an informal dataset that has not been associated directly with a mathematical equation. In this study, MATLAB Simulink^®^ R2016a edition (The MathWorks, Inc.) was employed to undertake neural networking. 

Formulations were derived using variables of 14% *w*/*v*–16% *w*/*v* of PF 127 and 8% *w*/*v*–20% *w*/*v* of PPF. Formulations were obtained using MATLAB^®^, determining combination integer matrices, in 1% *w*/*v* concentration increments within the variable range of PPF and PF 127. The concentrations of PEG-PCL-PEG polymer and all other reagents were kept constant during the design of the study. A total of 39 formulations were derived and synthesized using this software, as noted in Table 1. All formulations were evaluated for parameters of the temperature of gelation before bioprinting and the duration of drug release after bioprinting. This was then analyzed as a factor (Equation (1)), with the highest factor representing the optimized formulation matrix.

(1) $$Factor=\frac{tg}{bt}\times Rd$$

where *tg* represents the thermogelation temperature of the synthesized copolymer, *bt* represents body temperature, and *Rd* represents the drug release duration. Performance training of the neural network was evaluated by the mean square error and regression analysis [20]. 

A designed network, using a multilayer feed forward-back propagation, containing an input, output, as well as a variety of hidden layers, refers to a system architecture where the gradient is computed for multilayer nonlinear networks. This backpropagation relationship thus uses large input and output datasets to determine a network mapping, thereby not requiring a definite mathematical equation to undertake the modeling. This gradient descent algorithm using backpropagation is classified as the Widrow–Hoff learning rule, using multiple-layer networks, with various degrees of optimization to the algorithm. 

For the neural network to display training algorithms on the basis of lowest mean square error and highest accuracy correlations, a training percentage value of 70% was selected in the network. Validation of 15% was undertaken, measuring the network generalization, thereby terminating training when generalization of the network stops improving. A testing percentage of 15% was selected, resulting in no effect on training parameters and providing an indication of an independent measure of performance during and after training of the network. Thus, this complies with 100% evaluation split into 3 categories of network priority. The algorithm employed in a study depends on the complexity of variables and desired strategic outcome of modeling. In this study, we used 3 types of algorithms, such as Levenberg–Marquardt, Scaled Conjugate Gradient, and Bayesian Regularization. The algorithm that obtained the best training results was employed for the ANN study [20]. 

In terms of expressing data in the form of equation variables, the input to hidden layer *U* was expressed by:

(*U*) = (*W*)(*I*) (2)

*W*, representing the weight, and *I* the input. Each term of the hidden layer matrix can be explained as follows:

(3) $$Uj=\overset{n}{\underset{i=1}{\sum }}WiIi-\Theta$$

Θ, representing the associated bias. Optimization in the hidden layer using transfer functions was conducted. Nonlinear functions {log-sigmoid (logsig), hyperbolic tangent sigmoid function (tansig)}, and linear function (purelin) were undertaken to investigate the ability to achieve optimum results. Equations (4)–(6) were employed to understand the sequencing of optimization of the network:

*f*(*U*) = *u* (4)

(5) $$f(U)\;=\;\frac{1}{\left[1+e^{\left(-u\right)}\right]}$$

(6) $$f(U)\;=\frac{2}{\left[1+e^{\left(-2u\right)\;}\right]-1}$$

As a means of determining the effectiveness of the models, the determination coefficient (R^2^) and the mean square error (MSE) were employed as follows:

(7) $$\mathrm{MSE}\;=\;\frac{1}{n}=\;\sum_{i=1}^{n}{\left(Y\;response\;predicted-Y\;response\;experimental\right)}^{2}$$

(8) $$R^{2}=1-\frac{\sum_{i=1}^{n}{\left(Y\;response\;predicted-Y\;response\;experimental\right)}^{2}}{\sum_{i=1}^{n}{\left(Y\;response\;experimental-Y\;response\;mean\right)}^{2}}$$

The adaptation learning function employed was the gradient descent, with momentum weight and bias learning function. Optimization of the learning function also varied with the number of neurons, resulting in observational learning with greater percentage validity. Parameters of the number of epochs, minimum gradient, and Mu employed were evaluated at 10^2^, 1^−10^, and 0.01, respectively. 

Thermogelation analysis on the 39 polymeric ink design formulations, as seen in Table 1, were undertaken using a Modular Advanced Rheometer (ThermoHaake MARS Modular Advanced Rheometer, Thermo Electron, Karlsruhe, Germany), comprising a C 35/1° Ti sensor. A temperature range of 10–40 °C was conducted, using a cone and plate inertia of 1.721 × 10^-6^ kg m^2^, analyzing 5 mL of sample. The sample was analyzed in the range of 0–1.0 Hz, in the region of the shear independent plateau of the strain amplitude sweep stress (11). G’, representing the effects of elastic energy (storage modulus), and G’’, representing the effects of viscous energy (loss modulus), were evaluated. The point of thermogelation occurred when the fluid nature of the gel (G’’) transitioned to a semi-solid composition (G’), being subjected to an increase in temperature, over constant sinusoidal oscillation. 

### 2.4. 3D Design of the Bioprinted Pseudo-Bone Drug Delivery Scaffold

**Figure 1:**
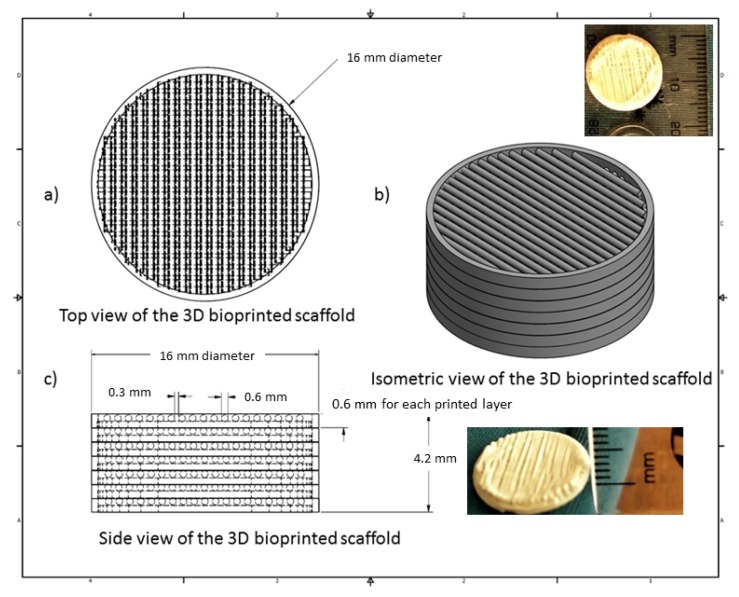
Computer-aided design (CAD) specification of the optimized 3D bioprinted scaffold.

The 3D bioprinted scaffold was designed using Autodesk Inventor^®^, 3D computer-aided design (CAD), for precise fabrication prototyping of the polymer-based biomaterial. The scaffold was designed as a cylindrical implant, with dimensions comprising 16 mm radius and a height of 4.2 mm. After generating a Stereolithography (STL) file on Inventor^®^, this file was imported to EnvisionTEC Visual Machines software, thereby converting to a Borland Package Library (BPL) file for bioprinting processing. Design of internal features and uniform slicing of the design was then undertaken on this software, printing a strand diameter of 600 µm and creating an inner structure pattern between layers at 30 °. The inner structural printing pattern of 30 ° was not designed in the CAD model, instead, it was implemented in the EnvisionTEC Visual Machines software as per the desired printing conformation. A needle tip of 0.8 mm diameter was used for printing the scaffold, which was set at an optimum 80% offset. This resulted in an average height of 0.6 mm printing diameter, with a deviation of +/− 60 µm per given strand. The deviation was due to drying of each strand between layers, which resulted minimally in the designed size. The designed scaffold, with a total number of 7 layers, is depicted in Figure 1. 

A 3D Bioplotter^®^ (EnvisionTEC GmbH, Gladbeck, Germany) was employed, using a pressure and temperature regulated syringe, with parameters optimized at 1.0 bar of pressure, speed at 1 mm/s, and syringe temperature regulated at 20 °C. The temperature of the printing platform was maintained at 40 °C. The transfer height and needle offset were set at 5 mm and 0.5 mm, respectively. Pre-flow delay, post-flow delay, and time between layers were set as 0, 0, and 120 s, respectively. The low pressure and speed of printing provided sufficient time for the structure to solidify, thereby promoting accuracy and scaffold platform building to occur. This technology allows the development of any object to be printed, provided the appropriate uniform viscosity is maintained throughout. Figure 1 illustrates the CAD design of the 3D bioprinted scaffold model. 

### 2.5. Chemical and Thermal Evaluation of the 3D Bioprinted Pseudo-Bone Scaffold

Nuclear magnetic resonance (NMR) was undertaken on the 3D printed scaffold using a Bruker AVANCE II 500 MHz (Bruker Avance Biospin, Germany) instrument. Deuterated chloroform (DCl_3_) was used to dissolve the scaffold, evaluating the sample at 25 °C. 

Thermogravimetric analysis was undertaken using a TGA 4000 thermogravimetric analyzer (PerkinElmer Inc, Massachusetts, USA) over a temperature range of 30–900 °C. This was undertaken at a ramping rate of 10 °C/min, under inert conditions, with a purge rate of 20 mL/min of nitrogen. A sample weight of 10 mg was used, evaluating the percentage degradation of the 3D bioprinted scaffold. The 1st derivative was obtained after analysis of the thermogram, detecting the point of inflection for analysis. This peak indicates the point of the greatest rate of change of the 3D bioprinted scaffold, with most significant weight loss observed. 

### 2.6. Morphological Analysis of the 3D Bioprinted Pseudo-Bone Scaffold and Rheological Evaluation of the Polymeric Ink 

Scanning electron microscopy (SEM) analysis was undertaken to confirm the pore architecture of the 3D bioprinted scaffold as well as to determine the accuracy of bioprinting parameters in relation to morphological characteristics between all 7 layers of the 3D scaffold. The 3D bioprinted scaffold sample was prepared by sputter coating on an aluminium spud, employing an EPI sputter coater (SPI Module TM sputter-coater and control unit, West Chester, PA, USA). The sample was then analyzed using an FEI ESEM Quanta 400 F (FEITM, Hillsboro, OR, USA) electron microscope, with an electron acceleration charge of 20 kV, producing high-resolution images of the 3D bioprinted scaffold. 

The viscoelastic behavior of the polymeric ink was evaluated using a Modular Advanced Rheometer (ThermoHaake MARS Modular Advanced Rheometer, Thermo Electron, Karlsruhe, Germany) comprising of a C 35/1° Ti sensor. Rheological measurements were evaluated at 10–40 °C, using a cone and plate inertia of 1.721 × 10^−6^ kg.m^2^. 0.5 mL of the sample was examined over a range of 0–1.0 Hz, falling within the shear independent plateau of the strain amplitude sweep stress [12]. The effects of elastic energy (storage modulus or G’) and viscous energy (loss modulus or G”) were observed after subjecting the sample to sinusoidal oscillation. 

### 2.7. In Vitro Drug Release Evaluation on the Designed 3D Bioprinted Pseudo-Bone Drug Delivery Scaffolds. 

All 39 bioprinted scaffolds (n = 3) were evaluated, employing a dialysis membrane (MWCO: 1.2 kDa) immersed in phosphate buffer solution (PBS, pH 6.8). Samples were evaluated in an orbital shaker incubator (LM-530-2, MRC Laboratory Instruments Ltd., Hahistadrut, Holon, Israel) at 37.5 °C, 50 rpm. One milliliter of sample was removed at each time point from the buffer and replaced equally with new buffer, which was conducted over a period of 30 days. Release samples were then analyzed for simvastatin concentration using a UV spectrophotometer at wavelength 238 nm (IMPLEN Nanophotometer^TM^, Implen GmbH, München Germany). This was undertaken using a 10 times dilution factor of path-length 0.1 mm [21]. 

### 2.8. Textural Analysis of the Human Clavicle Bone and 3D Bioprinted Pseudo-Bone Scaffold 

Matrix hardness (MH) and matrix resilience (MR) analysis, employing a textual analyzer (TA.XTplus, Stable Microsystems, Surrey, UK) under parameters of temperature at 37.5 °C and pressure of 1 atm, were undertaken on a healthy human clavicle bone (obtained with ethical waiver clearance) and thereafter on the area of the bone that was fractured and treated with the 3D bioprinted scaffold. A steel flat tip probe of 2 mm diameter was used for MH determination, and a steel cylindrical probe of 50 mm diameter was employed for MR evaluation. The clavicles were induced with a 4 mm diameter fracture in the region between the cervical fascia and the area below the conoid tubercle [22]. This was undertaken using a 4mm punch and dye apparatus, with a hydraulic pressure of 0.6 MPa. The fracture-induced human clavicle bone was then tested after incubation at 37.5 °C for 2 h, following hydration of the scaffold with 2 mL of PBS at the fracture site, with evaluation of the properties of matrix hardness and resilience on the bone thereafter.

## 3. Results and Discussion 

### 3.1. Design and Optimization of the 3D Bioprinted Pseudo-Bone Drug Delivery Scaffold Employing Artificial Neural Networks

**Figure 2:**
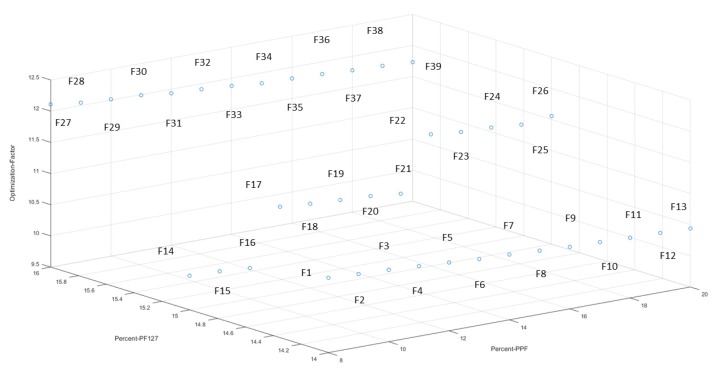
3D representation of the artificial neural networks (ANN) design formulations, reflecting the percentage of PPF and PF127, with the optimization factor for each formulation.

The 3D bioprinted scaffolds, evaluated as 39 design formulations using MATLAB^®^ software, comprised of variables of PPF (8% *w*/*v*–20% *w*/*v*) and PF127 (14% *w*/*v*–16% *w*/*v*), as presented in Table 1. These design scaffold formulations were studied in response to duration of release of simvastatin and the degree of thermogelation of the polymeric ink formulation. Figure 2 reflects the formulation compositions and response factor from each design, using a 3D Simulink design graph. It was observed that with incremental increases in the concentration of PPF at constant PF127 levels, a comparatively greater concentration of simvastatin was released for the formulations. This can be attributed to the increasing incompatibility created by the hydrophobic chain regions of PPF encapsulating simvastatin, a biopharmaceutics classification system (BCS) class 2 drug, at higher concentrations. It was also observed that, as the PF127 variable increased, the scaffolds biodegraded over an extended duration due to stronger gelation of the scaffold with increased PF127 concentration, further controlling the release rate of the loaded drug. This slower sustained release effect of PF127 in the formulation is desirable for implantable systems [23]. Furthermore, the formulations also demonstrated a decrease in gelation temperature as the concentration of PF127 was increased, as observed in Figure 4.

Providing these inputs in the software, Equation (1) was employed in determining the variable concentrations for the optimized formulation, thereafter training these inputs using ANN. The 546 number data set involved in the study was undertaken by varying the number of neurons in the hidden layer, using the sigmoid symmetric transfer function and using 3 different training functions for developing the model. The optimum network was derived using performance indicators of error function and R^2^ values. A variation in the number of neurons in the hidden layer is an essential component in ANN. The network thus becomes underperforming or highly entangled to sort, when the number of neurons is too high or low. Thus, a region between 6 and 16 neurons was investigated and considered an efficient model for optimum results. The optimum number of neurons after testing was found to be 10, thus producing the lowest mean square error and highest regression values for various training models.

For training of the network, the feed-forward backpropagation method was employed. Using the Levenberg–Marquardt, Bayesian Regularization, and Scaled conjugate gradient training networks, the training network that resulted in the lowest error functions (Equation (7)) and the highest regression value (Equation (8)) was evaluated. After much training and evaluation of input data, the Levenberg–Marquardt training function was observed to be the most effective algorithm employed using the sigmoid (tansig) function. Table 2 reflects the results obtained from the training algorithm and parameter performance observed. 

**Figure 3:**
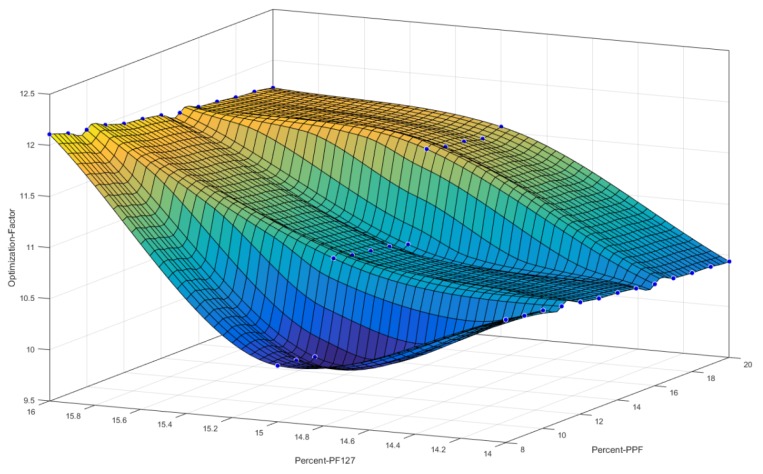
3D representation of the designed polymeric ink formulations using a cubic function surface plot, with the highest point on the surface plot representing the optimum polymer concentrations.

**Figure 4:**
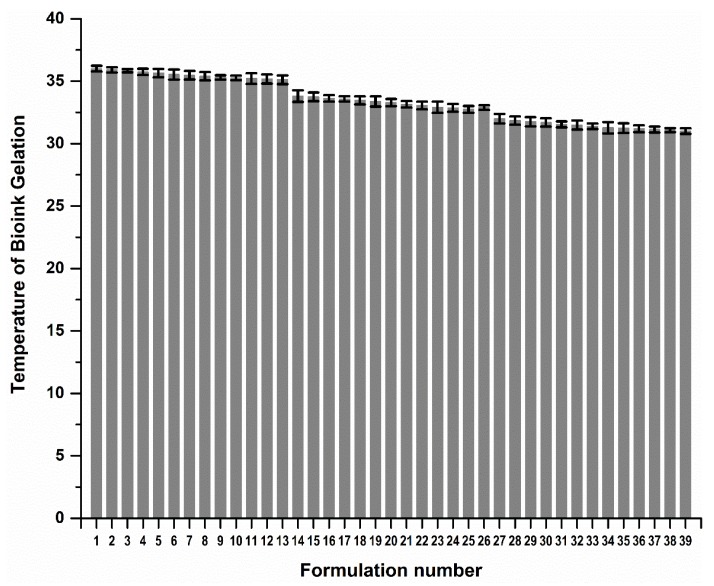
Gelation temperature of the 39 polymeric ink design formulations prior to 3D printing.

Figure 3 reflects a 3D cubic function of optimization parameters using a surface computed plot. The optimized formulation with the greatest factor, representing an optimum ratio of release duration and polymeric ink thermogelation, was found to be 14% *w*/*v* of PPF and 16% *w*/*v* of PF 127. This optimized formulation composition was thus selected as the superior formulation specification. Figure 4 represents the thermogelation temperature of the 39 polymeric ink formulations, highlighting a decrease in gelation temperature as the concentration of PF127 was increased due to the characteristic nature of the polymer. 

### 3.2. Chemical and Thermogravimetric Analysis of the Optimized 3D Bioprinted Pseudo-Bone Scaffold

**Figure 5:**
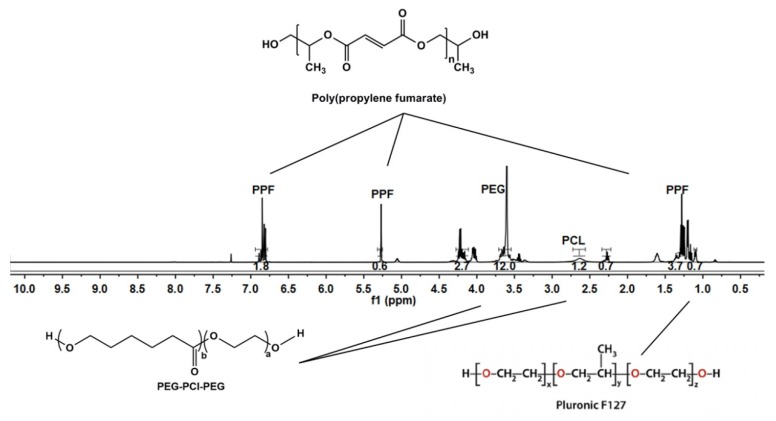
NMR analysis of the 3D bioprinted pseudo-bone scaffold, reflecting chemical shifts and copolymeric composition.

NMR analysis was undertaken on the 3D bioprinted scaffold, evaluating each chemical component in the formulation. As visualized in Figure 5, the broad signal peaks in the region of 3.5 ppm and 3.65 ppm represent the −(CH_2_)− functional groups present in PEG, with PCL functionalities of −OCCH_2_− and −CH_2_OOC− in the regions of 1.6 ppm and 2.2 ppm, respectively. Evaluating peaks responsible for PPF, it was evident that the defining functionalities of −HC=CH− in the region of 6.75 ppm remained intact in the PPF backbone structure. The −CH_3_− functionality of PF127 was identified in the region of 1.1 ppm, with further evaluation reflecting no chemical shifting of this functionality of protons in the backbone of PEG-PCL. The peaks observed in regions 1–1.3 ppm can thus be attributed to the −CH_3_− groups present in PPF and PF 127, respectively, responsible for chemical shifts from the parent compounds. Peaks for PF 127 were also identified in the region of 3.4 ppm, reflecting protons of individual functional groups. Minor peaks of PPF, not evident in the 3D bioprinted scaffold, was suggestive of successful copolymeric blending interaction, with the end groups of the PPF polymeric chain implicated in the interaction [24,25,26]. 

**Figure 6:**
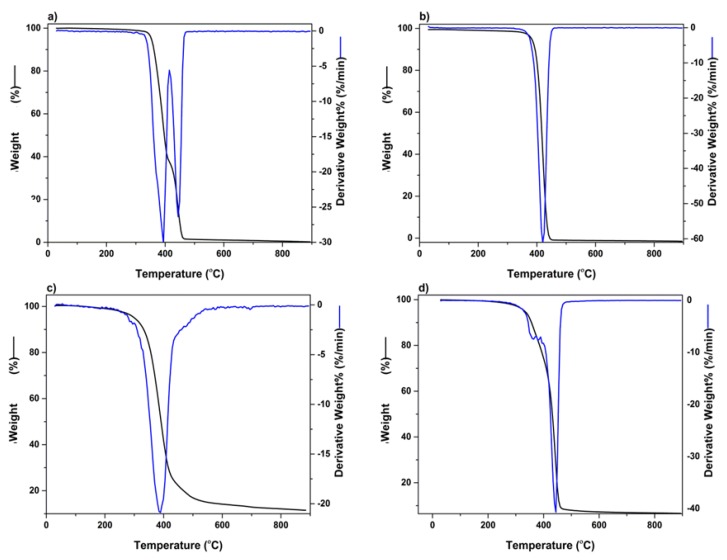
TGA analysis undertaken in the region of 30 °C to 900 °C for (**a**) PEG-PCL-PEG, (**b**) PF127, (**c**) PPF, and (**d**) 3D bioprinted scaffold.

The thermogravimetric analysis was undertaken to determine the temperature range, resulting in the greatest weight loss experienced in the 3D bioprinted scaffold, after being exposed to temperatures of 30–900 °C. Figure 6a represents polymer PEG-PCL-PEG, producing a double point of inflection, with the maximum degradation for PEG and PCL chains observed in the region of 387 °C and 448 °C, respectively. An initial percentage of degradation below 100 °C was attributed to the release of moisture in the sample, due to the hygroscopic nature of the polymer. PF 127 demonstrated significant biodegradation in the range of 412 °C, with PPF reflecting substantial weight loss at 379 °C, as seen in Figure 6b,c, respectively. Figure 6d depicts the 3D bioprinted pseudo-bone scaffold. As observed, the higher point of inflection at 448 °C indicated that the scaffold possessed greater thermal stability compared to individual polymers, possibly due to properties of increasing interfacial adhesion in the scaffold matrix. 

### 3.3. Morphological Analysis Undertaken on the 3D Bioprinted Pseudo-Bone Scaffold

**Figure 7:**
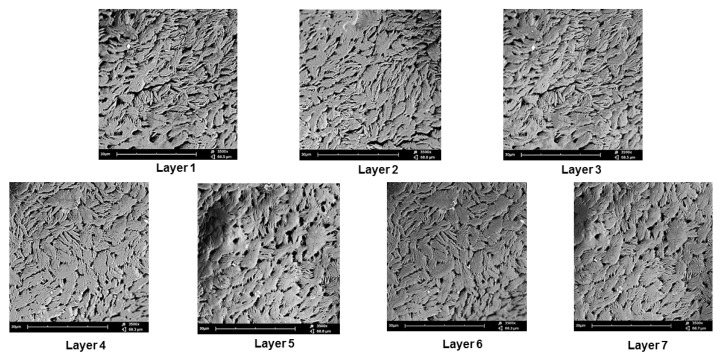
SEM analysis of the 3D bioprinted scaffold at 3500 times magnification, demonstrating the microarchitecture and inner porous nature of the 7 layers of the designed 3D scaffold matrix.

Scanning electron microscopy was undertaken on the 3D bioprinted scaffold for determining the microarchitectural design according to the CAD bioprinting parameters. The morphology was investigated employing electron microscopy at an average of 3500 times magnification. As seen in Figure 7, each layer of the scaffold reflected a similar porosity configuration, with uniform intercalated threads of fibrous 3D printing, bioengineered for cell growth within the porous network. This configuration further allows easy diffusion of tissue medium through the scaffold matrix. Printing under low-pressure and low-speed parameters, thus, allows for maximum consistency and uniformity in the microarchitectural design of the 3D scaffold. As observed in Figure 7, the intercalated “rope-like” nature with porous network architecture can be identified with multiple sites of scaffold printing, bonding between each designed layer. Macrostructural analysis undertaken after 3D printing confirmed a 600 µm strand diameter, with a deviation of +/− 60 µm per given strand. This can be attributed to air pressure accumulation during 3D printing, as well as, drying rates between each layer, once printed. A slight change in temperature could also affect the drying rate of each layer, thus contributing to the slight deviation of strand diameter thickness. 

**Figure 8:**
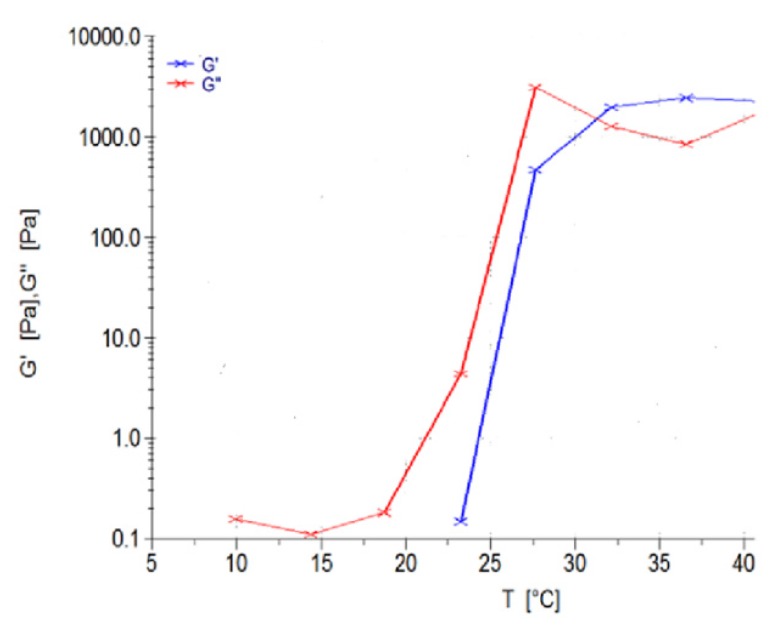
Rheological evaluation of the polymeric ink in relation to change in temperature.

The polymeric ink was evaluated with respect to change in temperature at a constant applied force, evaluating the change in temperature, as seen in Figure 8. G’ is described as the measure of deformation energy, referring to the elastic, solid properties of the polymeric ink that would adhere to the fracture of the bone. In contrast, G’’ is the measure of the viscous and deformation energy used and lost in the polymeric ink over a given temperature range. As seen in Figure 8, G’ begins at 23 °C, starting to form a semi-solid gelling property, and thereafter at 32 °C, completely switches to the elastic phase property, remaining above G’’ throughout the evaluation of the sample. It was observed that the viscosity of the sample gradually increases above room temperature, ± 25 °C, maintaining a higher elastic phase property than the liquid state at body temperature conditions.

Thus, we can determine that the polymeric ink has substantial thermo-responsive properties, with a significant change in modulus due to temperature variations. At controlled storage temperature of 10–20 °C, only viscous modulus is present in the range of 0.1–0.3 Pa, allowing ideal 3D printing to occur. When the higher temperature is reached, the polymeric ink increases strength by 45,000 times, allowing swelling and adhesion to occur (from 0.1 Pa to 4500 Pa). As body temperature is reached by the polymeric ink, the gel forms a solid, semi-elastic substance, thereby gradually releasing drug in a controlled, sustainable manner.

### 3.4. In Vitro Analysis of the Designed 3D Bioprinted Drug Delivery Scaffolds

The 39 designed 3D scaffold formulations were analyzed for their simvastatin release behavior. It was observed that as PPF polymer (8% *w*/*v*–20% *w*/*v*) was increased in percentage in the formulation, greater release of simvastatin from the scaffold over 24 h was observed. This can be attributed to the ester linkage of PPF, accounting for hydrolysis of the polymer into biocompatible and excretable degradation products of fumaric acid and propylene glycol, with associated release of the drug from the matrix [27]. 

**Figure 9:**
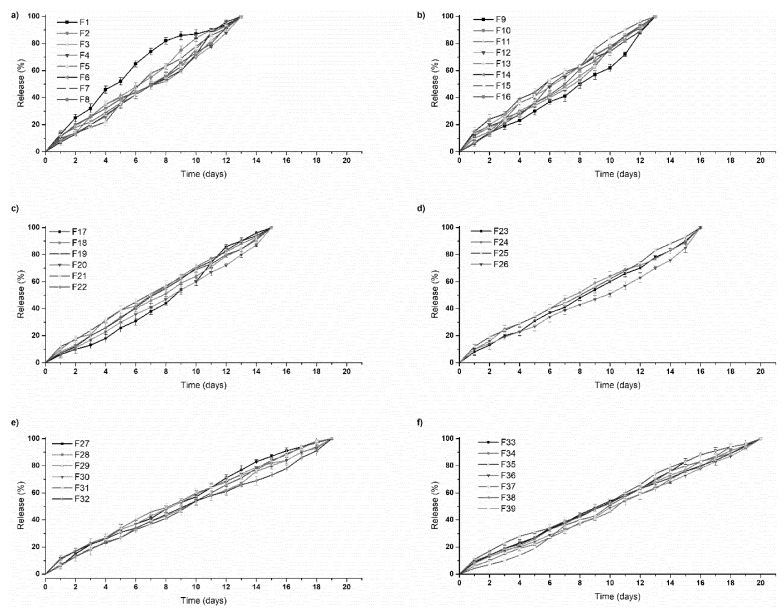
In vitro simvastatin release analysis of the designed 3D bioprinted drug delivery scaffolds. (**a**,**b**) formulations 1–16, released drug up to 13 days; (**c**,**d**) formulations 17–26, released up to 16 days; (**e**) formulations 27–32, released up to 19 days; and (**f**) formulation 33–39, released over a duration of 20 days.

**Figure 10:**
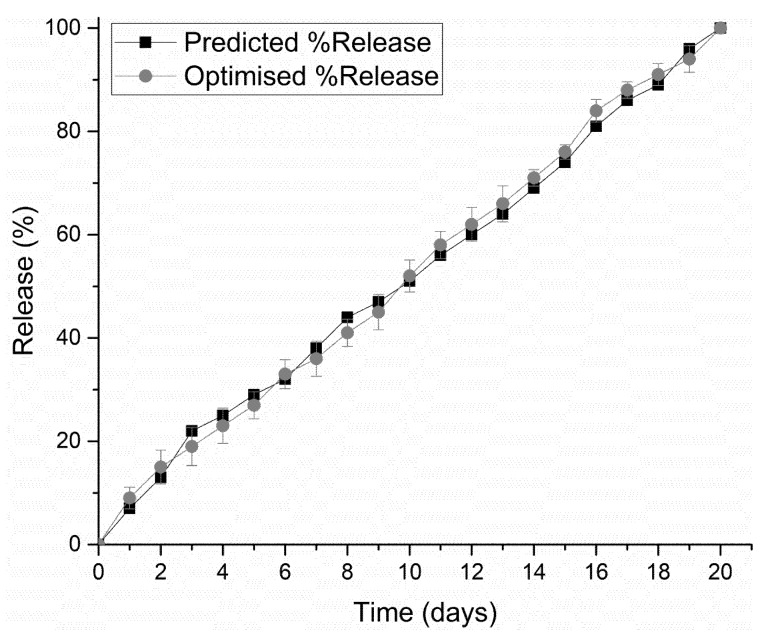
Correlation of in vitro simvastatin release analysis of the optimized 3D bioprinted scaffold with predicted release kinetics using ANN modeling.

PF 127 was incorporated at concentrations 14% *w*/*v* to 16% *w*/*v* in the formulations. It was observed that as the concentration of PF 127 was increased in the 3D scaffold, release of the loaded drug from the formulation was gradually slowed. This could possibly be explained in terms of increasing the amphiphilic nature of the 3D scaffold, resulting in an improved controlled release profile. The thermogelling capabilities of the system also contribute to the controlled release potential of the system by preventing particle aggregation, balancing hydrophilicity, surface roughness, and surface charge [23]. As observed in Figure 9, formulations 1–16 released drug up to 13 days, formulations 17–26 up to 16 days, formulations 27–32 up to 19 days, and formulation 33–39 up to 20 days. All formulations demonstrated near zero-order release kinetics. The optimized 3D bioprinted scaffold formulation, resulting in the highest factor of response (most sustained drug release), was thus synthesized incorporating 14% *w*/*v* PPF and 16% *w*/*v* PF127. The optimized 3D bioprinted scaffold displayed a controlled release of simvastatin over a 20-day duration, as seen in Figure 10, with significant correlation to the predicted release kinetics ascertained using ANN. It can be further emphasized that the morphological configuration in terms of the specialized shape and internal architecture significantly influenced the release kinetics and biodegradation of the 3D bioprinted drug delivery scaffold. It can be concluded that the optimized 3D bioprinted scaffold possessing highly specific design features of microarchitectural pores and uniform bioprinted filaments of specific dimensional properties has favorable controlled release kinetics in vitro, which demonstrated good correlation to the release predicted by the ANN model. 

An advantage of employing 3D printing over these parameters holds premise for the incorporation of cellular/biological materials to be integrated for future studies. Another advantage of this delivery system is attributed to the hydrophobic chain regions of PPF encapsulating a class of BCS 2 drugs, at higher concentrations. As PF 127 concentration increased, the scaffolds biodegraded over an extended duration of time, resulting in stronger gelation of the scaffold with increased PF 127 concentration. This can also be seen in Figure 9, as further controlling the release rate of the loaded drug was observed. Thus, a major advantage in the design was the slower sustained release effect of PF 127 in the formulation, being desirable for implantable systems. 

### 3.5. Matrix Analysis of the 3D Bioprinted Scaffold in Fracture-Induced Human Clavicle Bones 

**Figure 11:**
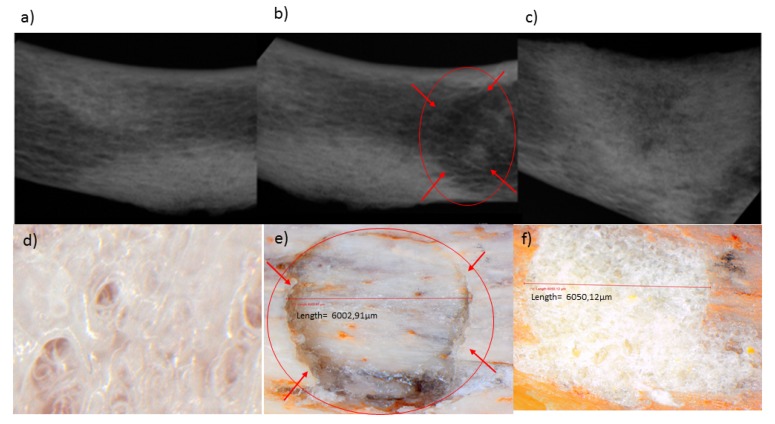
X-ray images of the human clavicle bone (**a**) before fracture, (**b**) after fracture, and (**c**) after treatment with the 3D bioprinted scaffold, respectively. (**d**) Light microscope image at 24 times magnification of the 3D bioprinted scaffold immersed in phosphate buffer solution, (**e**) human clavicle bone induced with a fracture, representing missing bone fragments, (**f**) human clavicle bone tested after incubation at 37.5 °C for 2 h, demonstrating sealing of the induced fracture site, with properties of matrix hardness and resilience comparable to original bone properties.

Mechanical properties of 3D scaffolds are essential in relation to their site-specific application [28,29]. The butterfly fracture was induced using a 4 mm punch and dye apparatus, using a hydraulic pressure of 0.6 MPa, under standard conditions (temperature of 25 °C and pressure of 1atm). A steel flat tip probe of 2 mm diameter was used to determine the MH and a steel cylindrical probe of 50 mm diameter used for determination of MR. The MH and MR were evaluated employing a texture analyzer resulting in values of 18.61 N/mm^2^ and 9.48%, respectively, for the human clavicle before fracture. Figure 11a–c depicts an X-ray image of the human clavicle bone before fracture, after fracture, and after treatment with the 3D bioprinted scaffold, respectively [29,30,31]. Following fracture, the missing bone mass was observed in the X-ray image (Figure 11b), as well as in Figure 11e, which is circled in red. After inducing the fracture, the 3D scaffold was applied at the site of defect, with immersion in PBS, and incubated at 37 °C for 20 days. The bone was then evaluated for MH and MR. It was found that a MH of 18.45 N/mm^2^ and MR of 9.33% were observed at the site of the fracture, thus possessing similar results to the non-fractured bone. Figure 11d represents a light microscope image at 24 times magnification of the 3D bioprinted scaffold immersed in PBS. After incubation of the scaffold in PBS at 37.5 °C for 2 h at the fracture site, microscopic visualization revealed significant filling of the fracture site, as observed in Figure 11f (depicting the architecture of the bone and the scaffold sealed sites). These values of MH and MR further exemplifies the unique properties of the 3D bioprinted pseudo-bone scaffold to fill in fracture sites in bones, thus promoting greater adhesion of bone and restoration of damaged bone to its intended mechanical integrity. 

## 4. Conclusions 

A 3D bioprinted pseudo-bone drug delivery scaffold was designed to mimic the morphology, matrix strength, and matrix resilience of healthy human bone. The 3D bioprinted scaffold was developed using computer-aided design (CAD) software, with further optimization of the design formulations employing MATLAB^®^ software and artificial neural networks. Polymers employed for formulating the 3D bioprinted scaffold consisted of polypropylene fumarate (PPF), free radical polymerized polyethylene glycol- polycaprolactone (PEG-PCL-PEG), and pluronic (PF 127). Simvastatin was incorporated into the 3D bioprinted scaffolds to further promote bone healing and repair properties. The 3D bioprinted scaffold was characterized for its chemical, morphological, mechanical, and in vitro release properties for evaluation of its behavior for application as an implantable scaffold at the site of fracture. The ANN-optimized 3D bioprinted scaffold demonstrated favorable properties as a controlled release platform, displaying sustained drug release over 20 days. The 3D bioprinted scaffold thus promoted contact adhesion between fractured/damaged bone using a human clavicle bone model, promoting the formation of a pseudo-bone matrix within the fractured site. Future investigations to be reported include in vitro cell culture studies, with biocompatibility evaluation on the 3D bioprinted scaffold, with completion following in vivo analysis. In in vivo studies in New Zealand, Albino rabbit model is being undertaken to confirm the degree of bone repair and regeneration promoted by the 3D bioprinted scaffold. It can thus be concluded that the significant research undertaken will demonstrate promising results for future research endeavors in bone healing and repair. 

## Figures and Tables

**Table 1:** Design specifications of the 3D bioprinted scaffold formulations using MATLAB Simulink^®^.

Formulation Number	PPF (% *w*/*v*)	PF-127 (% *w*/*v*)
1	8	14
2	9	14
3	10	14
4	11	14
5	12	14
6	13	14
7	14	14
8	15	14
9	16	14
10	17	14
11	18	14
12	19	14
13	20	14
14	8	15
15	9	15
16	10	15
17	11	15
18	12	15
19	13	15
20	14	15
21	15	15
22	16	15
23	17	15
24	18	15
25	19	15
26	20	15
27	8	16
28	9	16
29	10	16
30	11	16
31	12	16
32	13	16
33	14	16
34	15	16
35	16	16
36	17	16
37	18	16
38	19	16
39	20	16

**Table 2:** Training functions undertaken for optimization of the design formulations.

Training Algorithm	Mean Square Error (MSE)	Regression Function (R^2^)
Levenberg-Marquardt	≤0.1	9.99
Bayesian Regularization	≤0.1	9.82
Scaled conjugate gradient	0.7	9.14
